# Riding in the Cars

**Published:** 1860-01

**Authors:** W. Weybridge


### RIDING IN THE CARS.

One of the most important promotives of health is the getting along smoothly in the world, and one of the ways of doing this, is to be habitually courteous and accommodating, and to "give a little." Don't stand up for all your rights. Do not exact the last cent due you in your dealings, under the deceptive plea, that you owe it to yourself to be just, and to the one dealing with you, to let him see that you will not countenance imposition. In our experience through life, we have found that generous men have about as good an idea of what is justice as any other class of people; for they are just enough to make allowances for the mistakes, forgetfulness, prejudices, misapprehensions and ignorance of their fellow-men. Hence if his grocer makes a mistake of a dollar or two in his own favor, he does not go off in a huff, and let him severely alone for the remainder of his life. If a poor man makes a purchase of him, and lacks a few cents, he does not refuse to let him have the article on the plea that it is wrong to give any one the opportunity of defrauding. If a neighbor in straitened circumstances borrows a. dollar, to be returned certainly on a fixed day and hour, and fails, he does not resolve that he will not notice him the next time he meets him, and that he will never help him again the longest day he lives.

Ah! there are very many of the opposite of this, such precise people! we hate their characters. They are a living lie to themselves, and a disgrace to humanity. The generous man, instead of going away in a rage, turns an eye of pity and consideration on the delinquent. He has the magnanimity to suggest a sufficient reason for the short coming. The grocer may have made a mistake in casting up his accounts at the close of a weary day's labor, (and it was just as likely to be against himself,) as who may not in making any addition? The poor man whose heart is oppressed with the care of a helpless family, may have forgotten the trifling difference of a few cents, in the more important question, where shall I get work to day to keep me and mine from starving? The borrowing neighbor may be on a sick-bed and alone, or he may have confidently relied on the promise of a rich man to pay his "little bill," without fail, and that was the last of it.

But surely we have gone a wool gathering! we have run off the track most decidedly, for we intended to commend to public notice as a sensible man and a benevolent, Mr. W. Weybridge of Medford, who, on the fifth day of October, eighteen hundred and fifty-nine, wrote an article which is quite as good as the one we published ourselves last summer, and which ran the round of the press in little or no time. We copy the article, premising that whoever follows the advice which "Mr. Brown" had wit enough to see was worthy of being printed, will be liberally paid for his consideration.

Mr. Brown: I will tell travellers how to ride in cars. Open your eyes. Find out where you are going. Be five minutes in front of time. Semper paratus. Get into an ample linen overcoat with pockets. Take sufficient money for your journey, then double it; take no trunk if you can help it; take "refreshments," quantum sufficit, from your wife's clean store-room; take her advice and take a kiss to season it; but do not keep the cars waiting. Buy your ticket at the office. Look out for your pocket-book and check your baggage. Give a kind word to your conductor; take your seat before the cars have got in motion. Let your position be as near the centre of the car as possible, for wheels are dangerous and noisy. Enter into easy conversation with your seat-companion. Draw him out; the dullest will have something to instruct or entertain you if you skillfully address him. If a lady, let her lead the way, or sit in silence. Do not read, but talk, or *think*. Be attentive to the aged; to the ladies. Have a "bon bon" for the child that cries behind you; and keep to your good rule of taking every thing with cheerful temper through the day. Eat not your "lunch" alone. The half is better than the whole. Wear still a smiling face; for this is "evangelical" and better than a sermon. "Keep your eyes open." Men are books that *are* books; here you have a chance to read them. There'll be plenty of sleeping in the grave. Be alive while you are alive; make others so. Avoid a window slightly raised, a door ajar; a "cold" comes in that way, and then a "cough," and then a "coffin." Let the cars *stop*, stone still, before you leave them. A leg is heavier than ten seconds of time; but life goes but too often, with the leg. Eye your baggage; help that lady also. Pay your hackman in advance, but walk if possible; you need the exercise. Transact your business promptly, honorably, judiciously. Behave as well in the stranger city as at home. Keep away from haunts of mischief. Read Proverbs 7th chapter, commencing at the 4th verse. Go read it now lest you forget it. Do not sacrifice water for wine. Pick up information, by scraps if you must, but be sure and get it. Hasten home as soon as possible; your wife is at the window. "Keep your eyes open," I repeat again; be a true gentleman in *every* place, and you will enter your dwelling wiser than you went out of it, and will not trouble the ears of the one "you left behind you" with "doleful groans" about the miseries of travelling, the ill manners of men, nor will you be likely ever to bring an action against a railroad company, or it one against you.

W. WEYBRIDGE.

